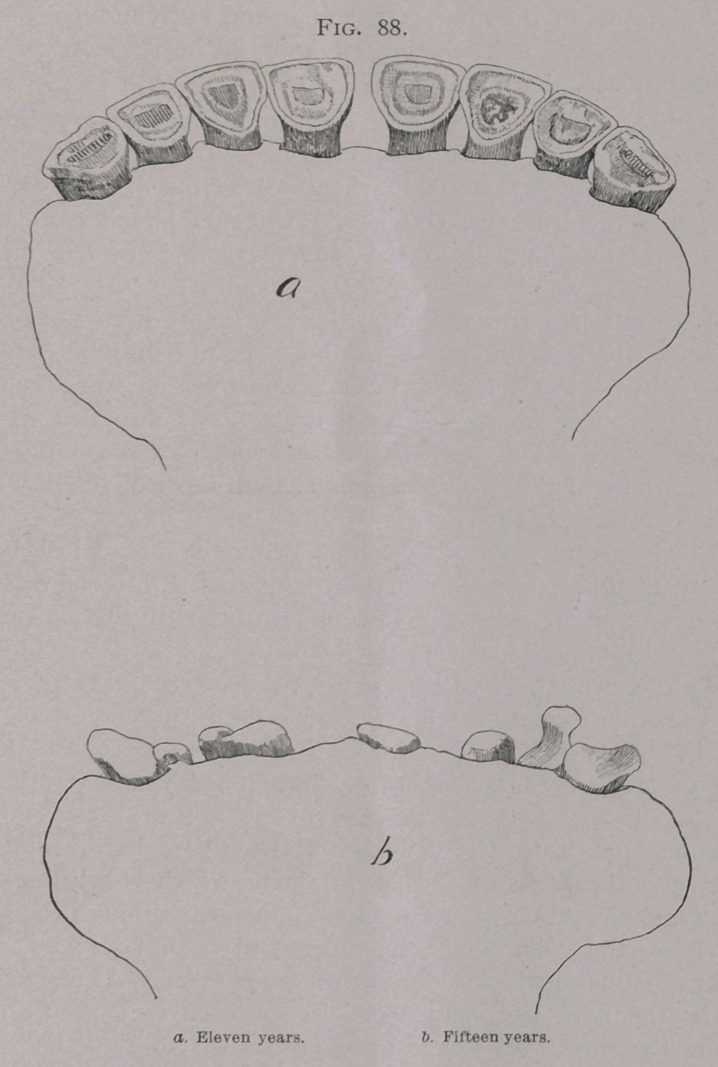# Age of the Horse, Ox, Dog, and Other Domesticated Animals

**Published:** 1891-09

**Authors:** R. S. Huidekoper

**Affiliations:** Vet.


### AGE OF THE HORSE, OX, DOG, AND OTHER DOMESTICATED ANIMALS.

By R. S. Huidekoper, M.D., Vet.

[*Continued from page 382.*]

### *AGE OF THE OX.*

The age of the ox has demanded much less study than that of the horse, on account of its shorter life, and the more limited time in which it is utilized for specific purposes. In a growing animal the evidences of its youth are unmistakable. Arrived at adult life, the difference of a year or two in its age is less important, in regard to its value, than it is in the horse, and later becomes even less so. The age of the ox is determined by the changes which take place in its dentition, and the wearing away of its teeth, and by the changes in the growth, form, and appearance of its horns.

### Dentition of the Ox.

The ox has thirty-two teeth, twenty-four molars, arranged as in the horse, in arches of six, on either side of each jaw, and eight incisors in the lower jaw, with none in the upper. In rare cases there are rudimentary molars (wolf teeth), but when these exist in the young mouth, they drop before the permanent dentition is complete. Tush teeth are not present.

Fig. 81.

Left Pincher Teeth of the Ox.

I. External faces.

II. Internal faces.

*aa.* Perment Incisors.

*bb.* Temporary Incisors.

Natural size.

### Incisors.

The upper jaw is devoid of teeth, but the intermaxillary bones are covered by a dense cartilaginous cushion and strong gum, which furnishes a resisting body to the incisors of the lower jaw, in the prehension of food.

In the lower jaw there are eight incisors, arranged like the ribs of a fan, on the spatula shaped arch formed by the extremity of the maxillary bone.

The incisors instead of being fixed solidly in their alveolar cavities, like those of the horse, are imbedded in them, on a layer of cartilage, which allows of a considerable amount of motion, and thereby, probably protects the cushion of the upper jaw from injury in seizing food, which is crushed rather than cut off.

The two middle incisors are known as *pinchers*, the next ones on either side as *first intermediate* teeth, the next as *second intermediate*, and the outside ones as *corner* teeth.

The two pinchers are slightly separated, on account of the cartilaginous symphysis of the maxillary bones in the ox ; this is much more marked in the first dentition. The other teeth touch each other by their extremities, and form a complete arch, but, from their shovel shape, are not in contact along their borders, as the wedge shaped incisors of the horse are.

The incisors are composed of a crown and a root, separated by a distinct neck giving them a somewhat shovel shape. The crown or free portion is flattened from above to below and becomes (Fig. 81.) thinner and broader at its anterior extremity.

The external or under face is convex in both directions, it is of a milky white color, and is striped with little longitudinal ridges and gutters, which become polished smooth with age. The internal or upper face is almost flat, but has a conical elevation, the base of which is directed towards the free border with which it merges, while its sides are bordered by small gutters. The internal border is convex and the external border is concave which gives the tooth a curved shape, and which indicates the side to which it belongs. The root is rounded, conical and yellow in color ; its extremity, in a young tooth, shows the opening of the dental canal.

### Structure.

The incisors have approximately the same structure as the tush tooth of a horse, they are composed of a dentine, with the free portion covered by a continuous layer of enamel. The enamel is thickest on the external surface and gradually disappears on the root. In the young tooth there is a large, simple, dental cavity filled with the dental pulp, but as the animal gets older, a dark yellow dentine is deposited, until the cavity is filled up, and the tooth ceases to grow. It is not pushed from its alveolar cavity, as the incisors of the horse are as the free portion is worn away.

Like the horse, the ox has two sets of incisors, the temporary, or milk teeth and the permanent ones. The milk teeth are distinguished from the permanent ones from their being smaller and narrower, their enamel is thinner and more transparent and they are more curved to the side. Their roots are very short, and are pushed out by the replacing permanent incisors.

The incisor scarcely reaches its full development when it commences to be worn by its contact, and constant friction, with food and with the cushion of the upper jaw. The wearing commences at the anterior border and removes the enamel towards the posterior part of the upper face; when it has completely removed the conical eminence and the lateral gutters, the tooth is said to be *leveled*, and the table is formed. From the almost horizontal direction of the incisor teeth, the wearing of their tables takes place in an oblique direction to their long axis.

The table at first is large, consisting of a plate of dentine, surrounded by a border of enamel and having in its centre a transverse line of dark yellow, made by the uncovered, later, deposit of dentine in the pulp cavity. As the tooth becomes smaller towards its root, the table becomes smaller and narrower, and the dental star becomes narrower, but it also becomes longer from in front to behind, until it forms a distinct square, as it is exposed by its posterior face from the oblique leveling.

As the incisors are worn away, they seem to separate from each other at the roots, as the narrower parts of the crowns are held apart by their cartilaginous beds, which do not atrophy, as does the maxilla of the horse, when the narrow wedge shaped roots are almost driven from their alveolar cavities.

When the teeth are worn down to their roots, the gum retracts, and shows the yellow stubs which are all that is left of the incisive arch. (Fig. 88 *b.*)

With the wearing away of the incisors, the dental arch loses its regular curve and becomes depressed in the middle.

### Molars.

The ox has, like the horse, six molars in each arch, on either side of each jaw. The arch is shorter, as the teeth are smaller. The first molar is very small and each increases in size to the sixth, but there is such a marked difference in the size, that the first three teeth occupy but the third of the arch, while the last three complete the posterior two-thirds.

In the ox, the molars have the same compound arrangement of enamel, as in the horse, filled up by dentine, and surrounded by a layer of cement; the latter often exists in great quantity, and is of a rich yellowish color. There seems to be a greater difference in the relative density of the substances, and the ridges of enamel stand out in sharper points.

As in the horse, there are three temporary molars, and six permanent molars.

According to Girard, the first temporary molar appears from the sixth to the twelfth day, following the eruption of the second and third molars, which are sometimes through the gums at birth, or appear immediately after birth.

The permanent molars make their appearance in the following order: 2d molar from twelve to eighteen months; 1st molar from twenty-four to thirty months; 3d molar at thirty-six months; 4th molar at eighteen months; 5th molar at twenty-four to thirty months; and the 6th molar at three years or later. When the rudimentary molar (wolf tooth) exists, it appears about the tenth month, and is driven out of its alveolar cavity with the appearance of the 1st permanent (4th) molar at the age of two years.

Simonds claims, that the molars do not show in the calf at birth, and first make their appearance at the end of the first month. Simonds also differs slightly from Girard as to the eruption of the permanent molars, placing the appearance of the 4th molar at six months; the 5th at fifteen months; the 6th at two years. He evidently studied on very precocious cattle. Undoubtedly the eruption of the molars in cattle is variable, and owing to the trouble of examination in the living animal, its careful study has been neglected.

An annexed table shows the diversity of eruption in various subjects, it will be found to vary somewhat not only in the different races of cattle, but in the families of the same race, which are reared under different climatic conditions, and are nourished more or less liberally.

The periods of the animal's life, as indicated by the teeth, form the following natural divisions:

1st, Eruption of the temporary teeth;
2nd, Wearing of the temporary teeth;
3rd, Eruption of the permanent teeth;
4th, Leveling of the permanent teeth;
5th, Wearing away of the crowns.

### DETERMINATION OF AGE BY THE TEETH.

With the young calf to be slaughtered for veal the absolute age is usually less important than its condition of development and its weight, but may be the cause of serious legal question and require the most acute preception on the part of the expert to decide and testify in controversies between the suspected butcher and the rigid law.

### FIRST PERIOD.

*Eruption of the Temporary Teeth.*—The calf is sometimes born with no incisors, but usually the pinchers and 1st intermediate teeth have pierced through the gums. The 2d intermediate teeth appear about the tenth day, and the corner teeth seven to ten days later, but may appear as late as the thirtieth day. The incisors do not reach the same level and complete the arch until the fifth or sixth month. (Fig. 82).

FIG. 82.

Incisors of Calf.

*a.* External face. *b.* Internal face.

### SECOND PERIOD.

*Wearing of the Temporary Teeth.*—The leveling of the milk teeth is very variable, according to the food on which the calf is fed. In calves fattened for the butcher with milk, the wearing is slow, while in those that are put early to pasture, and are fed on dried forage, the leveling takes place much more rapidly; the pinchers are worn at their anterior borders at six months, and are leveled at ten months; the arch is broken in the centre, and loses its continuity at the 1st intermediate, at one year; 2d intermediate, at fifteen months; and, at the corner teeth at eighteen to twenty months.

THIRD PERIOD.

*Eruption of the Permanent Incisors.*

TWENTY MONTHS.—At this time the milk pinchers are replaced by the permanent ones, which protrude somewhat obliquely, but soon assume their natural position and are in place at two years. (Fig. 83.)

FIG. 83.

Two years.

*a.* External face. *b.* Internal face.

The crowns of the permanent pinchers, in this month, have not quite become free from the gums, the left hand tooth is some what in advance of the other, and its enamel just shows traces of use.

Two Years, Six Months.—Between two and one-quarter years to two years and nine months the 1st permanent intermediate teeth have accomplished their eruption. (Fig. 84.)

Fig. 84.

Two years, nine months.

*a.* External face. *b.* Internal face.

In this mouth the crowns of the 1st intermediate teeth are free from the gums, and the root of the 2nd intermediate (temporary) is pressed on and its position slightly displaced.

THREE YEARS, THREE MONTHS.—Between three years and three and one-half years the 2nd intermediate have replaced the milk teeth. (Fig. 85.)

FIG. 85.

Three years six months.

*a.* External face. *b.* Internal face.

In this month the second intermediate teeth have reached the level of the incisive arch and their enamel has commenced to be used.

FOUR YEARS.—Between three years and nine months and four years and six months the corner teeth are completely through the gums and the mouth is complete. (Fig. 86.)

FIG. 86.

*a.* External face. Four years. *b.* Internal face.

The permanent corner teeth have just replaced temporary ones, and are still in an oblique position, not having completely emerged from the gum.

FOURTH PERIOD.

*Leveling of the Permanent Teeth.*—At five years the pinchers have commenced to level.

At six years the pinchers are leveled, both pairs of intermediate teeth are nearly so, and the corner teeth are somewhat worn. (Fig. 87 *a.*)

At seven years the 1st intermediate teeth are leveled, the 2d intermediate are much worn, and the corner teeth have lost their enamel at the anterior extremities.

At eight years the entire tables are leveled, and the pinchers commence to show a concavity, which corresponds to a convexity of the cushion of the upper jaw. (Fig. 87 *b.*)

At nine and ten years this concavity extends to the intermediate teeth, the table of the pinchers is almost square, and the dental star of the pinchers and 1st intermediate teeth has become long and distinct. (Fig. 87 *c.*)

During this period, from six to ten years, the rounded arch formed by the incisors gradually loses its convexity until it almost forms a straight line. The teeth appear to separate and the gum shows between them.

FIFTH PERIOD.

*Wearing away of the Crowns.*—From this time on there is a progressive change in the shape of the teeth, the crowns become worn down with more or less rapidity they diminish in size, the dental stars become larger and square, the teeth seem to separate, and the retracting gum leaves the yellow roots uncovered.

At ten years the dental star is square in the pinchers and the 1st intermediate and the corner teeth are leveled.

At eleven to twelve years the dental star is square in all of the teeth, which become triangular in shape and commence from this time on to be worn to stubs. (Fig. 88 *a.*)

It must always be borne in mind, that the race of the animal and the character of the food, produce great variations in the wearing of the teeth. Animals fed on hard forage, and those fed on brewers grains will have their teeth worn down much more rapidly than those fed on the prepared food of the ordinary dairy.

DETERMINATION OF AGE BY THE HORNS.

The horns of cattle, rising more or less gracefully from the frontal bones, were undoubtedly intended for weapons of offense

FIG. 87.

*a*. Six years. *b*. Eight years. *c*. Ten years.

and defense. All breeds of cattle are provided with horns, except that known as the angus or polled angus, which was indigenous to the northern part of Great Britain from the earliest historical times, but of which we have no trace in prehistoric deposits.

FIG. 88.

*a*. Eleven years. *b*. Fifteen years.

Among all varieties of cattle individuals may be devoid of horns; they are known as "mulley," or if deprived of their horns artificially (dehorned-dishorned) are called "polled." Polling has been done by the Hindoos for over the last two thousand years, without ever showing trace of producing hereditary results.

The horns are symmetrical in shape, and when there is any noticeable difference in the length, size or curve of the horns, it may be assigned to some previous injury, disease or accidental cause.

According to the character, habits and surroundings of the various races and individuals, we find the horns more or less modified in size, shape and strength.

In the semi-wild races, like those of Asia, Hungary, Spain, Texas and South America, the horns may attain enormous size, five feet in length; while in the most civilized, domesticated races, like the Durham, Dutch and Channel Island cattle, the horns tend to diminish in size, and may be but a few inches long.

The bull has strong, stout, short, straight horns, dense in structure, which seem to be as much points of hold for his massive, heavy head, as actual weapons in times of warfare; the female has longer, sharper, more delicate horns, designed to use in emergencies. The steer has horns, which are a compromise between those of the two sexes, longer than those of the bull and larger than those of the cow.

The horns have for a basement two *cores*, or conical bony projections of porous structure, richly supplied with blood vessels, and containing air cells which communicate with the sinuses of the frontal, occipital and maxillary bones.

The cores are covered by a dense fibrous vascular membrane, from the outer face of which, corresponding to the chorion of the skin, the horns grow. The horns themselves are conical tubes more or less curved, consisting of concentric layers of epithelial growth.

Soon after birth the calf shows two little, hard, rounded points at either side of the frontal bone, which slowly emerge from the skin. At eight or ten days the point is through the skin and shows the color which the horn will have later; at three weeks a distinct little flexible horn has appeared. At five or six months the horn has commenced to curve on its long axis and assume the direction it will have later. Up to this time and during the first year the horn is covered by an epidermic prolongation of the skin, similar to the covering of the hoof of the foal at birth, but by the twelfth to fifteenth month this covering has dried and scaled off, leaving the natural, shining, tough surface of the horn proper.

In the second year the horns start a fresh growth, and a small groove is found encircling it, between the substance secreted the first year and that which developed in the second.

During the third year a similar activity in growth takes place and a second groove is found marking the line between the two years' growth. These two grooves or circular furrows around the horn are not well marked and have been frequently overlooked and all trace of them disappear as the animal becomes older.

From three years on, the growth of the horn is marked by a groove or furrow, much deeper, and so distinct that they show between them a decided elevation or "ring" of horny substance, which forms an accurate basis for estimating the age of the animal.

In an animal over three years of age we count all of the horn beyond the first groove as indicating three years, and add one year to its age for each groove and "ring" which is present toward the base of the horn.

The grooves are always better marked in the concavity of the horn than on the convex surface. In feeble, ill-nourished animals they are but slightly marked.

Many causes, however, tend to diminish the value of the "rings" and grooves in the estimation of age. In "show" cattle and in herds of cattle kept for show, the horns are frequently sandpapered, scraped and polished to give them the fine appearance of delicate texture, which with that of the other integument, indicates the similar condition of the mammary gland for secreting milk and of the connective tissue for forming fat. Dealers scrape the horns to destroy the evidences of age in the animals which they have for sale. In old cows there is an atrophy of growth and an apparent contraction of the base of the horn, the rings, and grooves are much less distinctly marked and may be indistinguishable.

In the first four years the teeth are the most valuable indications of age; from four to ten years the horns furnish the more accurate signs, and after ten years a careful comparison of both is required to determine approximately the number of years which have passed.

### TABLE OF ERUPTION OF THE TEETH IN THE OX.

| | SIMONDS. | | GIRARD. | OTHER AUTHORITIES. | | AVERAGE. | |
|---|---|---|---|---|---|---|---|
| | Race and other causes favoring development | Race and other causes retarding development | | Minimum. | Maximum. | In precocious animals. | In common animals. |
| **Temporary** *Incisors.* | | | | | | | |
| Pinchers. | | | At Birth. | Before Birth. | 3 days. | At Birth. | At Birth. |
| 1st Intermediate. | | | At Birth. | Before Birth. | 5 days. | " | " |
| 2nd Intermediate. | | | 5th to 9th day. | Before Birth. | 12 days. | 5 days. | 12 days. |
| Corners. | | | 13th to 19th day. | Before Birth. | 35 days. | 12 days. | 18 days. |
| *Molars.* 1st. | After Birth. | 1 mo. | 6th to 12th day. | At Birth. | 1 mo. | 6 to 12 days | 18 to 30 days. |
| 2nd. | At Birth. | Earlier. | Earlier. | " | Few days. | At Birth. | After Birth. |
| 3rd. | At Birth. | Earlier. | Earlier. | " | " | " | After Birth. |
| **Permanent** *Incisors.* | | | | | | | |
| Pinchers. | 1 yr. 9 mo. | 2 yrs. 3 mo. | 19 to 21 mo. | 15 mos. | 2 yrs. 3 mo. | 1 yr. 6 mo. | 20 mo. |
| 1st Intermediate. | 2 yrs. 3 mo. | 2 yrs. 9 mo. | 2½ to 3 yrs. | 2 yrs. 3 mo. | 3 yrs. | 2 yrs. 3 mo. | 2 yrs. 9 mo. |
| 2nd Intermediate. | 2 yrs. 9 mo. | 3 yrs. 3 mo. | 3½ to 4 yrs. | 2 yrs. 9 mo. | 4 yrs. | 3 yrs. | 3 yrs. 6 mo. |
| Corners. | 3 yrs. 3 mo. | 4 yrs. 9 mo. | 4½ to 5 yrs. | 2 yrs. 10 mo | 5 yrs. | 3 yrs. 9 mo. | 4 yrs. 6 mo. |
| *Molars.* 1st. | 2 yrs. 6 mo. | | 2½ to 3 yrs. | 2 yrs. 3 mo. | 3 yrs. | 2 yrs. 6 mo. | |
| 2nd. | 2 yrs. 6 mo. | | 12th to 18th mo. | 1 yr. 6 mo. | 2 yrs. 6 mo. | 1 yr. 6 mo. | |
| 3rd. | 3 yrs. | | 3 to 4 yrs. | 3 yrs. | 4 yrs. | 3 yrs. | |
| 4th. | 6 mo. | | 18 mos. | 6 mo. | 1 yr. 6 mo. | 1 yr. 6 mo. | |
| 5th. | 1 yr. 3 mo. | | 2½ to 3 yrs, | 1 yr. 3 mo. | 3 yrs. | 2 yrs. | |
| 6th. | 2 yrs. | | 3 to 4 yrs. | 2 yrs. | 4 yrs. | 2 yrs. 6 mo. | |

## Figures and Tables

**Fig. 81. f1:**
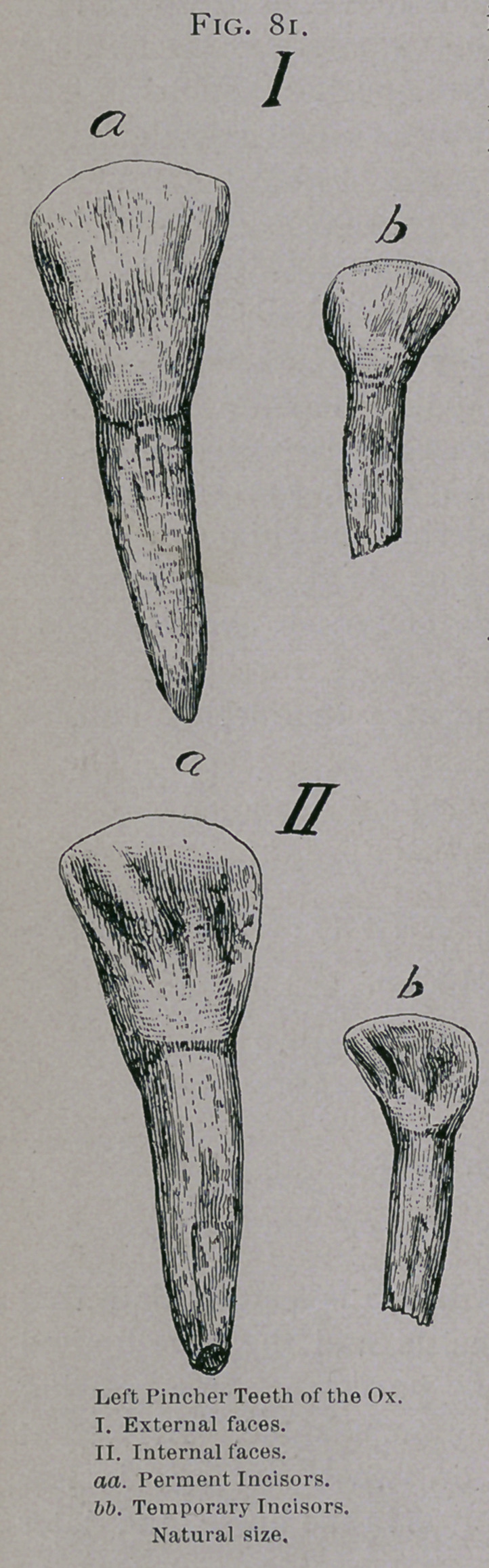


**Fig. 82. f2:**
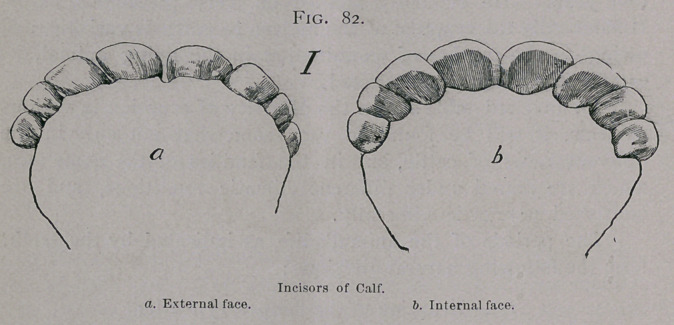


**Fig. 83. f3:**
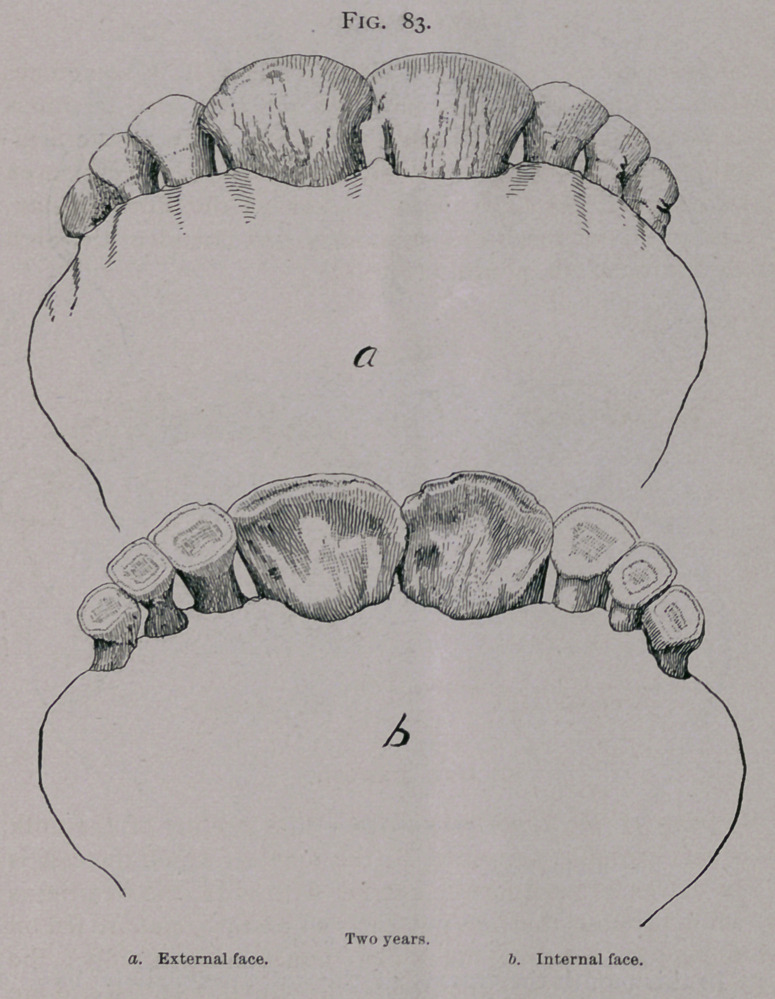


**Fig. 84. f4:**
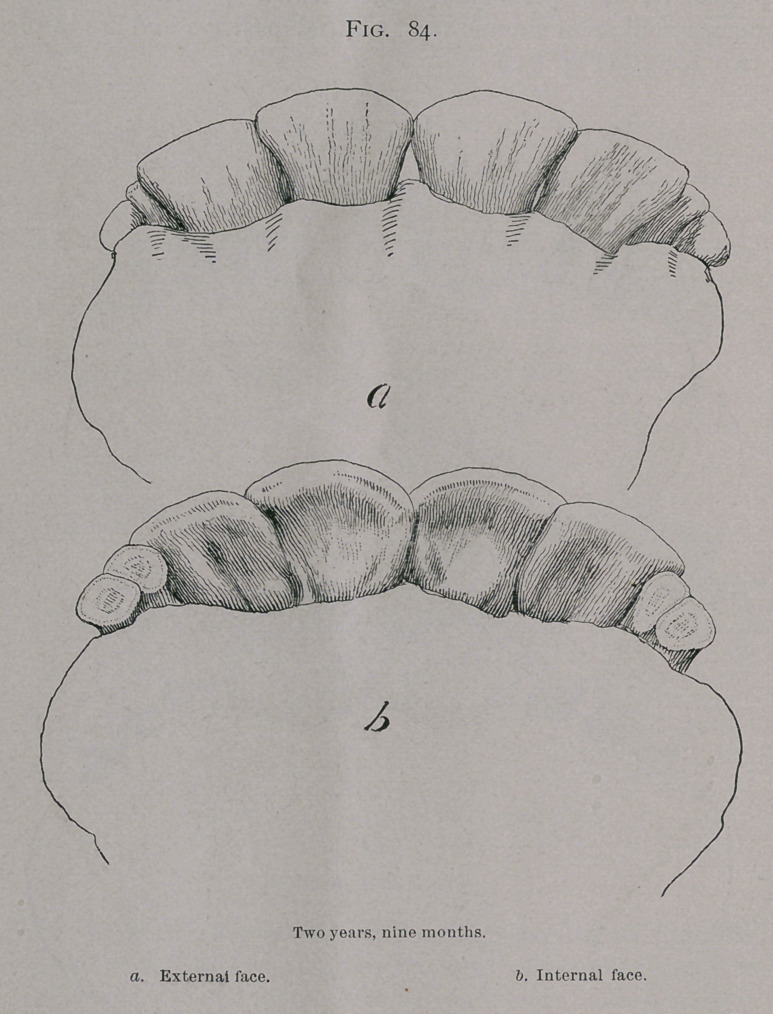


**Fig. 85. f5:**
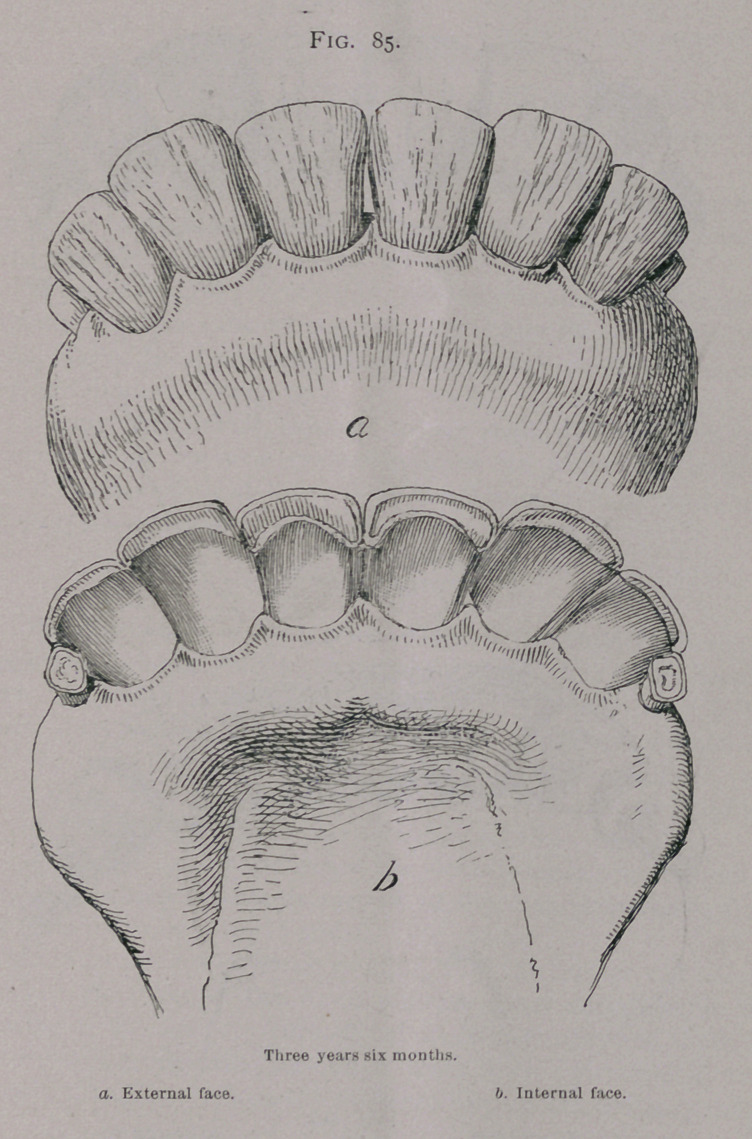


**Fig. 86. f6:**
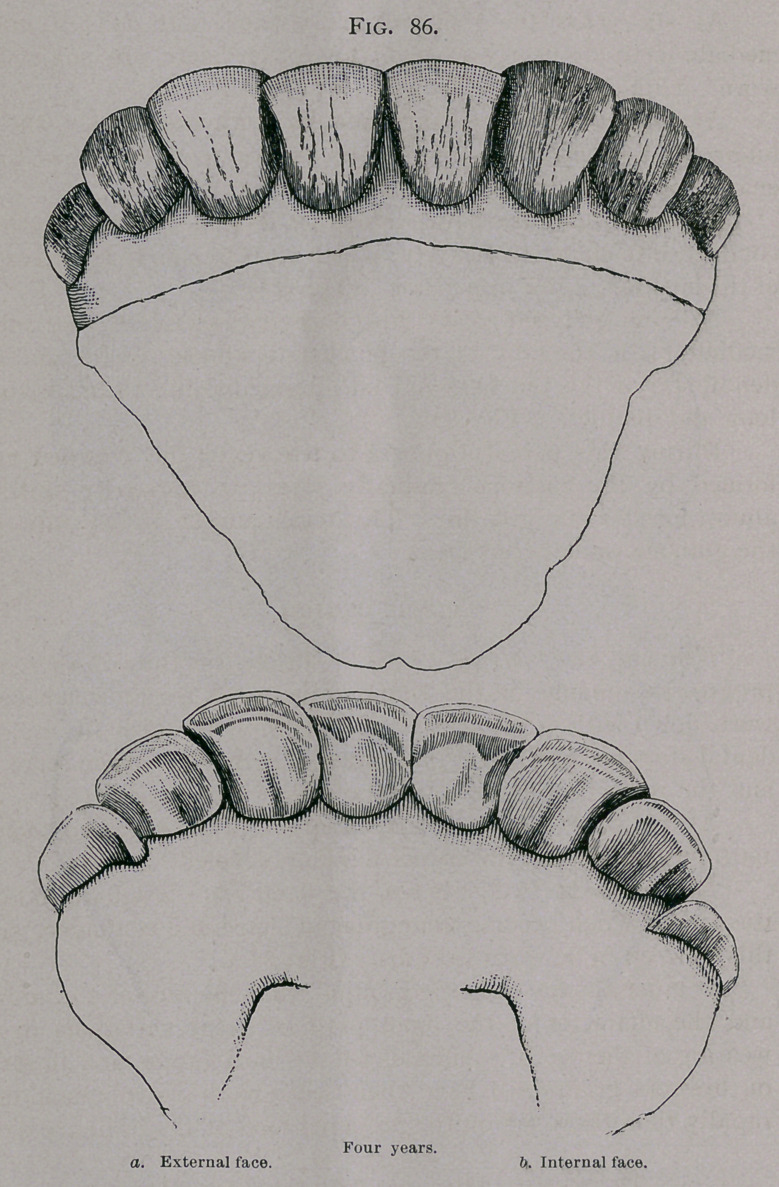


**Fig. 87. f7:**
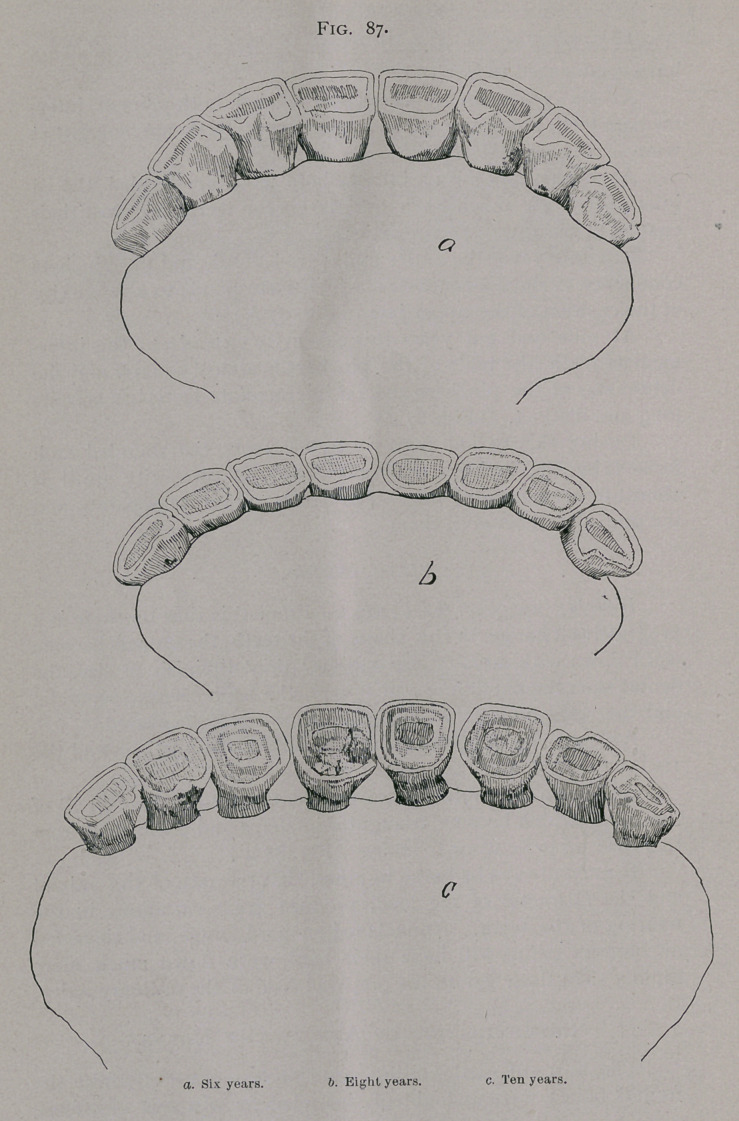


**Fig. 88. f8:**